# Identification, Characterization, and Expression of a Novel *P450* Gene Encoding CYP6AE25 from the Asian Corn Borer, *Ostrinia furnacalis*

**DOI:** 10.1673/031.011.0137

**Published:** 2011-03-28

**Authors:** Yu-liang Zhang, Mahesh Kulye, Feng-shan Yang, Luo Xiao, Yi-tong Zhang, Hongmei Zeng, Jian-hua Wang, Zhi-xin Liu

**Affiliations:** ^1^ Key Laboratory for Tropical Crop Biotechnology, Ministry of Agriculture, Institute of Tropical Bioscience and Biotechnology, Chinese Academy of Tropical Agricultural Science, Haikou, Hainan 571101, P. R. China; ^2^ Key Laboratory for Biological Control of Ministry of Agriculture, Institute of Plant Protection, Chinese Academy of Agricultural Sciences, Beijing, 100081. P.R. China; ^3^ Key Laboratory of Heilongjiang Microbiology, College of Life Sciences, Heilongjiang University, Harbin, Heilongjiang 150080, P. R. China; ^4^ College of Bio-safety Science and Technology, Hunan Agricultural University, Changsha, Hunan410128, P. R. China

**Keywords:** cytochrome P450, real-time PCR, bioinformatics

## Abstract

An allele of the cytochrome P450 gene, *CYP6AE14,* named *CYP6AE25* (GenBank accession no. EU807990) was isolated from the Asian com borer, *Ostrinia fumacalis* (Guenée) (Lepidoptera: Pyralidae) by RT-PCR. The cDNA sequence of *CYP6AE25* is 2315 bp in length and contains a 1569 nucleotides open reading frame encoding a putative protein with 523 amino acid residues and a predicted molecular weight of 59.95 kDa and a theoretical pI of 8.31. The putative protein contains the classic heme-binding sequence motif F××G×××C×G (residues 451–460) conserved among all P450 enzymes as well as other characteristic motifs of all cytochrome P450s. It shares 52% identity with the previously published sequence of *CYP6AE14* (GenBank accession no. DQ986461) from *Helicoverpa armigera.* Phylogenetic analysis of amino acid sequences from members of various P450 families indicated that CYP6AE25 has a closer phylogenetic relationship with CYP6AE14 and CYP6B1 that are related to metabolism of plant allelochemicals, CYP6D1 which is related to pyrethroid resistance and has a more distant relationship to CYP302A1 and CYP307A1 which are related to synthesis of the insect molting hormones. The expression level of the gene in the adults and immature stages of *O. furnacalis* by quantitative real-time PCR revealed that *CYP6AE25* was expressed in all life stages investigated. The mRNA expression level in 3^rd^ instar larvae was 12.8- and 2.97-fold higher than those in pupae and adults, respectively. The tissue specific expression level of *CYP6AE25* was in the order of midgut, malpighian tube and fatty body from high to low but was absent in ovary and brain. The analysis of the *CYP6AB25* gene using bioinformatic software is discussed.

## Introduction

Cytochrome P450s are a very large and diverse group of enzymes found in all domains of life. The *P450* genes are classified into thirty-six gene families based on the comparison of deduced amino acid sequences (Nelson 2005; Zhou and Huang 2002). P450 enzymes are found in virtually all insect tissues, where they are involved in processes that are vital for insect growth, development, and reproduction, including ecdysteroid and juvenile hormone biosynthesis and aspects of ecdysteroid degradation, as well as metabolism and detoxification of xenobiotics (Feyereisen 2005). In fact, as a reductionist's means of understanding resistance mechanisms in insects, over-expression of these genes has become a dominant criterion for identifying insecticide resistance—associated *P450* genes. Over-expression of *P450*s leading to increased metabolism of insecticides has been demonstrated in the housefly, *Musca domestica, CYP6A1* (Cariño et al. 1992 1994), *CYP6D1* (Liu and Scott 1998) and *CYP6A12* (Guzov et al. 1998), in *Drosophila melanogaster, CYP6G1* (Joussen et al. 2008) and *CYP6A2* (Amichot et al. 2004), and in *Plutella xylostella, CYP6BG1* (Bautista et al. 2007 2008). The study of Mao et al. (2007) reported *P450* gene function involved in xenobiotic metabolism (the natural plant insecticide, gossypol) in a lepidopterous pest. The isolation and characterization of specific insect P450s is a critical first step towards understanding the P450s involved in these important metabolic processes. Such studies have provided great insight into insecticide resistance, and insect development at biological and physiological level (Feyereisen 2005; Scott 1999).

The Asian corn borer, *Ostrinia furnacalis* (Guenée) (Lepidoptera: Pyralidae), is a major pest of corn in China (Zhai 1992). This insect, at times, causes significant economic damage to cotton by chewing holes in the leaves, tunneling into stems, branches, flower buds, flowers, and green bolls (Cao et al. 1994; Wu and Guo 2005). Although it is one of the major insect pests in China, no single *O. furnacalis P450* gene has been evaluated for its role in xenobiotic metabolism.

The experiments reported here were undertaken to identify and characterize a novel *P450* gene (*CYP6AE25*) from *O. furnacalis.* Real-time quantitative PCR was implemented to investigate the developmental and tissue specific expression of the gene in the adults and immature stages, to provide a base of gene expression data for future experiments on the regulation of this gene and its product. The identification of *CYP6AE25* in *O. furnacalis* could have potential applications in entomological research and control of insect pests in the field by plant-mediated insect RNAi technology.

## Materials and Methods

### Insect culture

*O. furnacalis* was reared in the Key Laboratory for Biology of Plant Disease and Insect Pests, Institute of Plant Protection, Chinese Academy of Agricultural Sciences, Beijing, China as described by Guo et al. (2010) at 25° C ± 1° C under a 14:10 L:D photoperiod and 70–80% relative humidity. Males and females were separated during the pupal stage, based on the morphology of abdominal terminal segments. After emergence, moths (15 females and 15 males) were caged in an open 4500 cm plastic container, given unlimited access to 10% sucrose solution, and allowed to copulate for a period of two nights. Mated females were allowed to lay their eggs on wax paper. The paper with egg masses was changed every other day, surface sterilized according to the method described previously by Xu et al. (2006) and transferred to a plastic box (15 cm in diameter and 6 cm in height). Between 12 and 24 h prior to egg hatching, a conventional artificial diet was offered by Song et al. (1999). After hatching of the eggs, 1^st^ instar larvae were collected and reared in individual vial until pupation. The 3 day old stages of 1^st^, 2^nd^, 3^rd^, 4^th^ and 5^th^ (last) instar larvae, pupae, and adults were used for experiments.

### Isolation of total RNA and synthesis of first strand cDNA

Total RNA was isolated from the different developmental stages of *O. furnacalis* using Trizol reagent (Invitrogen Life Technologies, www.invitrogen.com). About 20 mg of the insects were ground in a mortar and pestle in the presence liquid nitrogen. The ground powder was then thawed in 1 ml Trizol reagent. The total RNA was extracted and purified as per the manufacturer's instructions from DNase reagent kit (Takara, www.takara-bio.com). Finally, the total RNA (OD_260_/OD_280_ = 1.79) was dissolved in 40 µl DEPC-treated water and stored at -80° C until further use. The 1^st^ strand cDNA was synthesized using 2 µg of DNase-treated total RNA by RevertAid™ First Strand cDNA Synthesis Kit (Takara) with oligo(dT)-adaptors. The total volume of reverse transcriptional system was 25 µl. The reactions were performed according to the manufacturer's instructions and the reaction mixture was stored at -20° C until further use.

### The full-length cDNA amplification of *CYP6AE25*

The primers used in PCR were designed based on the reported sequences of conservative amino acid regions of P450 protein family in insects. The forward primer was 5′-TGGAAGGTNNTGCGKCAGAACCTRAC-3′ and the reverse primer was 5′-GCTTCWGGRTKYTTSGCCARYTC-3′. The expected nucleotide sequences including the middle fragment of *P450* gene was about 640 nucleotides. The amplification was performed with PrimeSTARTM HS DNA polymerase (Takara) under the following conditions: an initial denaturation at 94° C for 3 min, followed by 35 cycles of 94° C for 30 s, 50° C for 30 s, and 72° C for 1 min, and a final extension at 72° C for 10 min. There was 2 µl cDNA in the total volume of 25 µl. All the gene-specific primers were designed utilizing Primer Premier 5.0 (Premier Biosoft, www.premierbiosoft.com). The PCR products were separated by 1.5% agarose gel electrophoresis and stained with GoldView Dye. The band of the expected size (about 600 bp) was excised and purified using the Gel ExtractionMini Kit (TianGen Biotechnologies, www.tiangen.com). The purified products were cloned into a pMD19-T cloning vector (Takara) and transformed into DH5a competent cells (Transgen, www.transgen.com.cn). The positive clones were identified by PCR with the universal primers, M13-47 and RV-M, and sequenced in both directions (Invitrogen Life Technologies). The amino acid sequence deduced from the nucleotide sequence showed that it is related to the CYP6 family. The PCR fragment was therefore used as a probe to screen the full-size CYP6 gene. Using the Takara 5 ′RACE and 3′ RACE cDNA amplification kit (Takara): 5′ RACE primer: OfP450-5RACE-GSPl:5′-CATACACAAGCCCACCAACAT-3′ ; OfP450-5RACE-GSP2: 5′-CAGGGAAACTCTTCAGTCCTAA-3′, 3′ RACE primers: OfP450-3RACE-GSP1: 5′-GACAACTCCACAATAAGGGGCT-3′, OfP450-3′ RACE-GSP2: 5′-TTACTGGAGACAGCATAAGGAGCG-3′.

5′- and 3′-cloned fragments were obtained. The RT-PCR products were purified directly from bands excised from agarose gels and cloned into pGEM-T Easy Vector (Promega). Positive clones were sequenced.

### Bioinformatics software analysis

The cDNA sequence was compared with other lepidopteron insects. The translation into amino acid sequence was carried out with Swiss Prot database ExPASy Translate tool (au.expasv.org/tools/dna.html). The protein structure analysis was performed with ExPASy online ScanProsite (us.expasy.org/tools/scanprosite/). Signal P program analysis using N-terminal signal peptide sequence with online tools (www.cbs.dtu.dk/services/SignalP/), TMHMM analysis of the protein transmembrane domain with (genome.cbs.dtu.dk/services/TMHMM/), protein post-translational modification of glycosylation sites and phosphorylation site analysis with DictyOGlyc online software and NetPhos2.0 Server Analysis (www.cbs.dtu.dk/services/DictyOGlyc/; www.cbs.dtu.dk/services/NetPhos/); GOR secondary structure prediction (npsapbil.ibcp.fr/cgi-bin/npsa_automat.pl?page=npsa_sopma.html); ProtScale analysis of protein hydrophobicity with ExPASy protein analysis expert system (us.expasy.org/tools/protscale.html), three-dimensional structure of protein database PDB (Protein Data Bank) with ExPASy's 3djigsaw tool, three-dimensional molecular structure of the protein with RasMol software (bmm.cancerresearchuk.org/∼3djigsaw/) and functional analysis with Pfam domain structure (pfam.sanger.ac.uk/).

### Phylogenetic tree construction

Searching of similar sequences was performed using BlastP in the non-redundant protein sequences (nr) database of the NCBI website (www.ncbi.nlm.nih.gov/) from the GeneBank database, collected from 13 species of 17 cytochrome *P450* gene sequences, including five from mammals and rest from insects. Multiple alignments of the deduced amino acid sequences were conducted by Clustal X 1.81. A phylogenetic tree was constructed by MEGA3.1 (www.megasoftware.net/) applying the method of Neighbor- Joining (NJ).

### The developmental and tissue specific expression level of *CYP6AE25*

To compare the expression levels of *CYP6AE25* gene among the different developmental stages of *O. furnacalis,* Q-RT-PCR analysis was performed using comparative threshold cycle number (Ct) method (Li et al. 2001) on a Stratagene Mx3000P thermal cycler (Stratagene, www.stratagene.com) with the actin (GenBank accession no. GU301782) of *O. furnacalis* as a reference. The Ct was determined and used for comparative quantitative analysis. The total RNA for Q-RT-PCR was extracted from 3-d-old 1^st^, 2^nd^, 3^rd^, 4^th^ , and 5^th^ (last) instar larvae, pupae, and adults of *O. furnacalis* and treated with DNase as described earlier. In order to amplify the actin, the cDNAs were synthesized using random primers and the samples were used as PCR templates while the reaction conditions were strictly followed as per the cDNA Synthesis Kit (Takara). The primers were designed using the Primer 3.0 program (Premire Biosoft). The nucleotide sequences were: 5′-ACTGGAAGAAAAAGAATGTG-3′ for F1 and 5′ GGCTGCTGAAGTAGTAGAAA-3′ for R1 while the expected product size was 234 bp. The Primers used for amplification of actin gene were F2, 5′-TGGGACGACATGGAGAAGAT-3′ and R2, 5′-AGATAGGGACGGTGTGGGAG-3′ while the expected product size was 262 bp. The dilution curves generated by serial dilutions (1:20) of cDNA were used to calculate amplification efficiencies. Q-RT-PCR was carried out in 20 µl reactions system containing 2 µl of 1:30 diluted cDNA templates, 10 µl Brilliant®SYBR® Green QPCRMaster Mix (Stratagene) and 0.2 mmol of each primer. For each sample, reactions were performed in triplicates. Thermal cycling conditions were: 95° C for 10 min, 40 cycles of 95° C for 30 s, 60° C for 30 s, and 72° C for 30 s. After Q-RT-PCR, a melting curve analysis from 55 to 95° C was applied to all reactions to ensure consistency and specificity of the amplified product. The results of Q-RT-PCR were statistically analyzed with MxPro3.20 software (Stratagene). In addition, the amplification size was checked by running on agarose gel electrophoresis with the PCR product for each primer pair. The mRNA levels of *CYP6AE25* were normalized with actin levels in the same samples quantified in the same method, and three replicates obtained from four such independent assays were pooled to give the final relative mRNA levels in different developmental stages.

**Figure 1.**
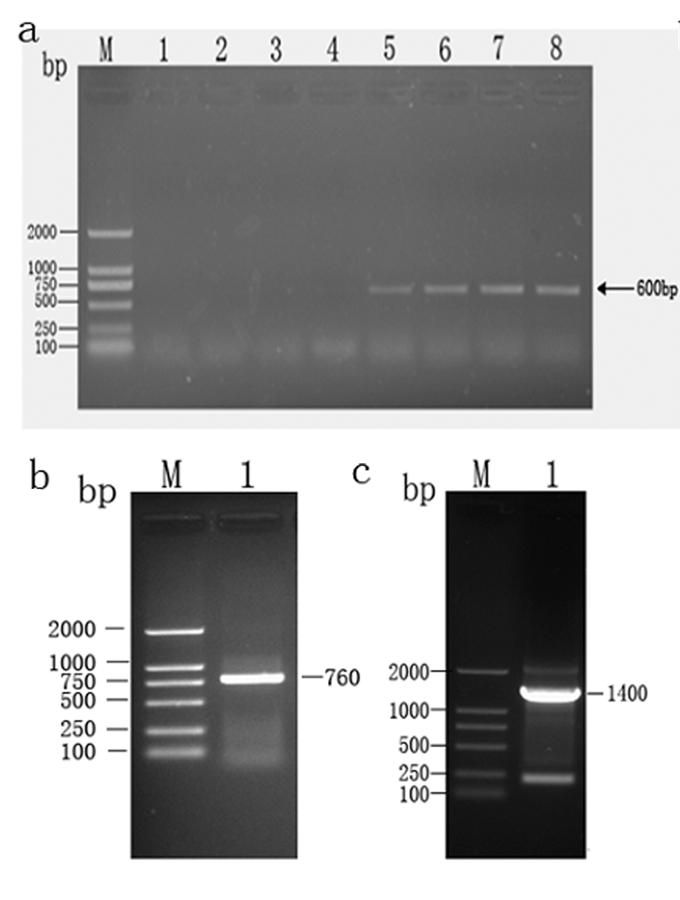
The full-length fragment of *Ostrinia furnacalis* P450 was obtained by RT-PCR and RACE technology. (a) The gradient PCR amplification of middle fragment of *CYP6AE25* gene by degenerate primers. Lane 1 to Lane 8 represents different annealing temperature *viz*., 48.0° C, 48.8° C, 50.3° C, 52.4° C, 55.3° C, 57.6° C, 59.1° C, and 60.0° C. (b) The 5′ RACE result of *CYP6AE25* gene. (c) The 3′ RACE result of *CYP6AE25* gene. High quality figures are available online.

The tissue specific expression of *CYP6AE25* from 3^rd^ instar larvae of *O. furnacalis* was detected by using RT-PCR with similar primer pairs and thermal cycle condition as described above.

## Results

### The identification and the characterization *of the CYP6AE25*

**Figure 2.**
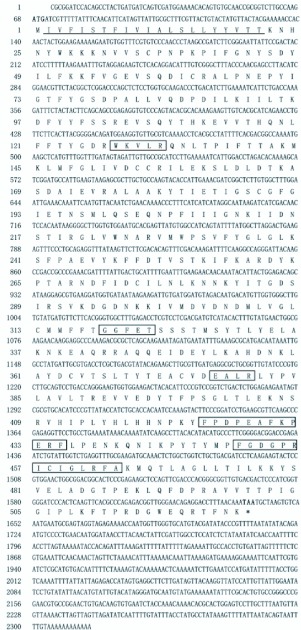
The full-length cDNA sequence of *CYP6AE25* and its deduced amino acid sequence. The start codon ATG is indicated with bold and the stop codon, TAA is indicated with bold and by an asterisk. The conserved regions of P450 enzymes including the heme-binding sequence motif FxxGxxxCxGxxxA are indicated by the boxed amino acid. The amino acid thickly underlined is the positions of transmembrane region, also belongs to region of Signal P prediction. High quality figures are available online.

Using the degenerate primers, positive clones were obtained (Figure 1a) and by overlaying the cloned sequences a full sequence of the P450 *CYP6AE25* gene was obtained (Figure 1b and Figure 1c). The cDNA sequence with the deduced amino acid below the nucleotide sequence (GenBank accession no. EU807990) is shown in Figure 2. The sequence analysis indicated that the deduced protein sequence shares high identity (52%) to *CYP6AE14* (GenBank accession no. DQ986461) from *H. armigera.* The sequence has been submitted to the P450 nomenclature committee and named as *CYP6AE25.* The sequence is 2315 bp in length with an ORF of 1569 bp, a 67 bp 5 'non-coding region (5′UTR) and a 676 bp with a polyadenylation signal of the 3 'non-coding region (3′UTR) encoding 523 amino acid residues with a predicted molecular weight of 59.95 kDa and a theoretical pI of 8.31. The start codon is located at positions 68–70 with the methionine as the starting amino acid while the termination codon is at positions 1637–1639. The deduced amino acid sequence of *CYP6AE25* shares a number of common characteristics with other members of the P450 superfamily (the boxed amino acids sequence). For instance, the classic hemebinding sequence motif F××G×××C×G (residues 451–460, in which C is the hemeiron ligand) that is conserved among most P450 enzymes, the cysteine which is present in all P450 sequences.

### Bioinformatic analysis

**Figure 3.**
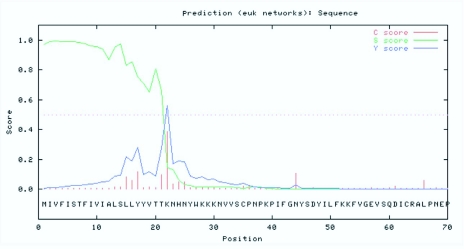
Signal P prediction of the amino acid sequence of CYP6AE25 from *Ostrinia furnacalis.* High quality figures are available online.

The transmembrane region prediction indicated that the cDNA of *O. furnacalis,* CYP6AE25, is a membrane protein with a 20-residue long transmembrane domain (residues 2–21 for CYP6AE25) located near the N-terminus. The comparative analysis of the fragment with the other insects *CYP6* gene family showed high homology. The homology for the lepidopteron insects such as *H. armigera* (CYP6AE14), *Bombyx mori* (CYP6AE) and *Depressaria pastinacella* (CYP6AE1) was 52%, 50% and 49%, respectively. The CYP6AE25 showed the half-life of 30 h, instability coefficient of 44.82 and chemical formula as C_27030_H_4224_N_690_O_777_S_25_. It has predicted that CYP6AE25 belongs to one of the instable protein family. The PSORT Prediction Analysis (Figure 2) supported the cytoplasmic protein function with N-terminal-like motif of the film containing XXRR retention signal KVLR, C-terminal-like motif of the film containing KKXX reservation signal AKNP. The Signal P analysis (Figure 3) revealed that the amino acid residue from the initiation of Met amino acid to 22 amino acids is predicted to be the signal peptide, and a possible cleavage site is located between 21^st^ Thr and 22^nd^ Tyr amino acid. Further experiments are needed at the cellular and molecular level for verification of the prediction. Signal peptide sequence in the first 4 to 20 of the 17 hydrophobic amino acid residues across the endoplasmic reticulum was integrated into the endoplasmic reticulum suggesting that CYP6AE25 may be involved in oxidative metabolism of exogenous compounds. It can be inferred that c *O. furnacalis* CYP6AE25 is related to the metabolism of plant secondary substances like DIMBOA. A spiral of its C terminal has characteristic XXG residues (-IFG-), C helix N-terminal sequence of WKXXR conservative (-WKVLR-), W, R residue and the role of heme propionic acid anions, with C helical N terminal connected to the meanderl area that is a bent area, their participation in the formation of activity sites and spatial location is rather conservative.

The E helix in both proteins with the formation of characteristic hydrophobic peaks in the spiral is located in the internal proteins. The spatial structure of the E helix C terminal has conserved residues of FG. (Nelson and Strobel 1988) reported that the DE helix loop exists between the large area involved in substrate binding. In the active site of P450 proteins is an important part of the I helix, GGFET of CYP6AE25 as an I spiral with characteristic structural units, the single-helix involved in substrate oxidation. The I helix of the C-terminal residues has a conservative Q form a corner into the J helix. The K spiral in all P450 proteins is simple to identify, with constant EXXR structural characteristics (-EALR-). Although the Meander region has characteristic secondary structures, its tertiary structure is rather conservative. Therefore, CYP6AE25 has a conserved sequence of YFPDPEAFKPERF, in which Y, F, P, D, E, A, K, R has a strong affinity for the polar water residues. The heme-binding domain with conserved structural units FG / SXGXH / RXCXGXXI / L / FA (FGDGPRICIGLRFA), both in terms of a sequence or a three-tier structure is the structure of the most conservative regions. Most of the P450 protein, containing 500 or so amino acids, in the C-terminal heme-binding domain has the structure of the domain. This domain determines whether a particular sequence is an important basis for P450 (Hasemann et al. 1995) closely followed by the L helix (-KMQTLAG-).

**Figure 4.**
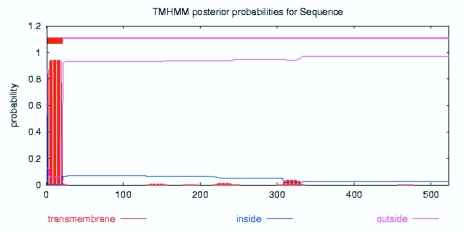
Transmembrane probabilities of the amino acid sequence CYP6AE25 from *Ostrinia furnacalis* with TMHMM analysis. High quality figures are available online.

**Figure 5.**
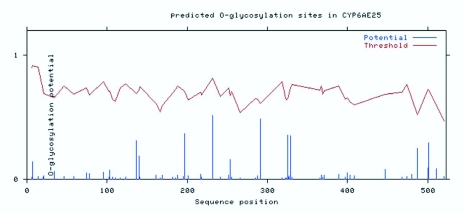
O-glycosylation site prediction of the amino acid sequence of CYP6AE25 from *Ostrinia furnacalis.* High quality figures are available online.

**Figure 6.**
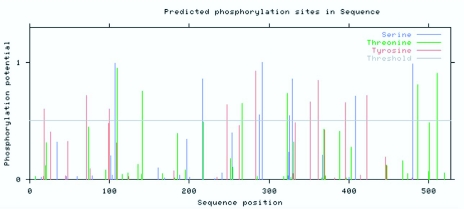
Phosphorylation site prediction of the amino acid sequence of CYP6AE25 from *Ostrinia furnacalis.* High quality figures are available online.

**Figure 7.**
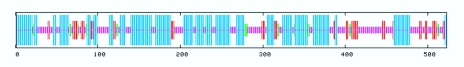
Secondary structure predication of the amino acid sequence of CYP6AE25 from *Ostrinia furnacalis.* High quality figures are available online.

The transmembrane domain is a membrane protein-lipid combination. It may play a role as a membrane receptor and may also be positioned in the membrane-anchored protein or ion channel proteins, etc. The use of biological software predicts that the transmembrane domain may be a correct understanding of protein structure, function and role in the cell membrane. TMHMM software analysis of the CYP6AE25 transmembrane domain showed that the N terminal of CYP6AE25 protein is a highly hydrophobic domain that contains a transmembrane domain, area number 2 from the peptide chain beginning to the end of the first 21 amino acids (Figure 4). DictyOGlyc projections (Figure 5) indicated the sugar-free glycosylation in CYP6AE25. NetPhos2.0 Server phosphorylation site analysis (Figure 6) of CYP6AE25 showed that there were 9 serine (Ser) phosphorylation sites, 6 threonine (Thr) phosphorylation sites and 9 tyrosine (Tyr) phosphorylation sites uniformly distributed throughout the polypeptide chain. The secondary protein structure analysis (Figure 7) of CYP6AE25 with GOR online tools showed that α-helix accounted for 47.23% and random coil accounted for 36.71%. These are the largest parts of the structural elements of CYP6AE25. The extended strand distributed throughout the protein accounted for 12.2%, and β-turn existed in small amount to only 3.8%.

**Figure 8.**
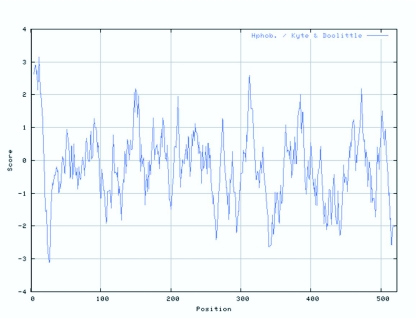
Hydrophobic character predication of the amino acid sequence of CYP6AE25 from *Ostrinia furnacalis.* High quality figures are available online.

Some amino acids are hydrophobic and tend to be repelled by surrounding water molecules and are embedded within the protein. This trend along with space, three-dimensional conditions and other factors that ultimately determine the formation of a protein folding into a three-dimensional conformation. ProtScale analysis (Figure 8) showed that the maximum hydrophobicity of CYP6AE25 was 3.15 and minimum was -3.1. The amino acids in the region 1 ∼ 10,130 ∼ 160, 300 ∼ 320, 380 ∼ 390, 460 ∼ 480 has strong hydrophobic properties and the majority of these regions is predicted to be α-helix, while the corresponding random coil region is predicted to have a very low level of hydrophobicity.

**Figure 9.**
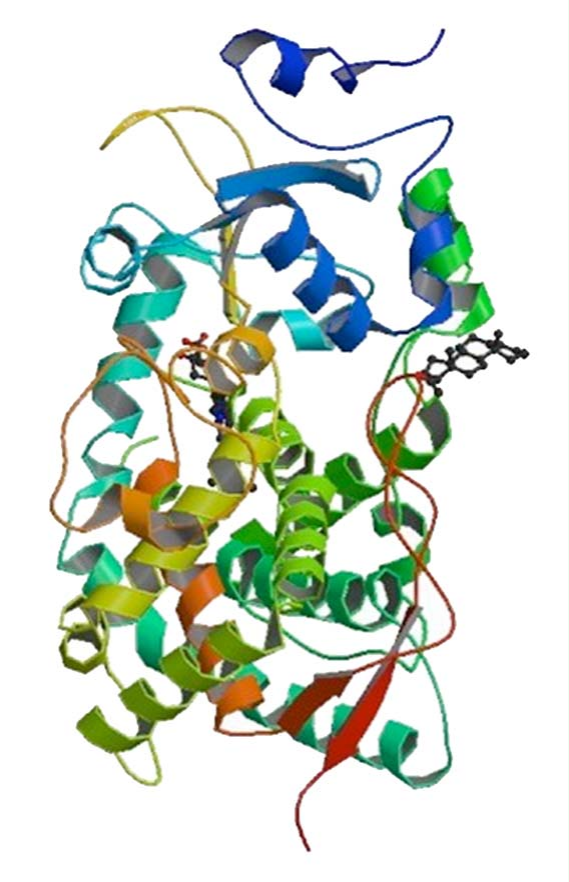
The comparative modeling of the tertiary structure of CYP6AE25 from *Ostrinia furnacalis.* High quality figures are available online.

The three-dimensional structure is the ultimate goal of protein structure prediction and it is necessary to fully understand protein function. Tertiary structure can be predicted from the amino acid sequences by using different techniques and methods, one of which is homology modeling, which compares the structure of protein sequences with known protein sequences to predict the protein structure. Using ExPASy's 3djigsaw tool to use the three-dimensional structure of the protein database PDB (Protein Data Bank) sequence homology, it was found that CYP6AE25 has highest homology with human liver CYP3A4 (33%). By using CYP3A4 protein crystal structure as a template for homologous modeling, high-level structure of CYP6AE25 was obtained using SwissPDBviewer and RasMol software (Figure 9). Insect P450 families have a contain a highly conserved P450 domain. The functional domain of cytochrome P450 monooxygenase components for CYP6AE25 was found by using Pfam-line analysis (Table 1).

### Phylogenetic relationship of *O. furnacalis* CYP6AE25 with other P450s

**Figure 10.**
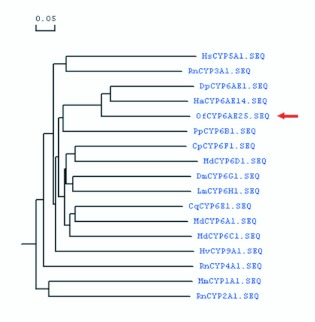
Phylogenetic relationship based on the amino acid sequence alignment of cytochrome P450s from various P450 families. Note: Mm: *Mus musculus* CYP1A1(NP_034122); Rn:*Rattus norvegicus* CYP2A1(AAH81848); Rn: *Rattus norvegicus* CYP3A1(NP_037237); Rn: *Rattus norvegicus* CYP4A1(AAA41038); Hs: *Homo sapiens* CYP5A1(AAF99269); Md: *Musca domestica* CYP6A1(AAA29293); Pp: *Papilio polyxenes* CYP6B1(CAA82732); Md: *Musca domestica* CYP6C1(AAA69818); Md: *Musca domestica* CYP6D1(AAA81513); Cq: *Culex quinquefasciatus* CYP6E1(BAA28946); Cp: *Culex pipiens pallens* CYP6F1(AAT72405); Dm: *Drosophila melanogaster* CYP6G1(NP_610743); Lm: *Locusta migratoria* CYP6H1(AAD39748); Hv: *Heliothis virescens* CYP9A1(AAC25787); Dp: *Depressaria pastinacella* CYP6AE1(AAP83689); Ha: *Helicoverpa armigera* CYP6AE14(ABI84381); Of: *Ostrinia furnacalis* CYP6AE25(ACF17813). High quality figures are available online.

The deduced amino acid sequence of CYP6AE25 consisted of all important motifs characteristic of the P450 enzymes, particularly the CYP6 family. Using MEGA3.1 software (www.megasoftware.net/). a phylogenetic tree was constructed using Neighbor-Joining (NJ) (Figure 10). The phylogenetic analysis of amino acid sequences from members of various P450 families revealed that CYP6AE25 has high phylogenetic relationship with CYPAE14 from *H. armigera* and CYP6AE from *Bombyx mori* but distant phylogenetic relationship with CYP6BF1v4, CYP6a8, and other members of CYP6 family. The phylogenetic analysis of amino acid sequences from members of insect CYP6 family revealed that CYP6AE25 has high homology, especially with the CYP6AE14 from *H. armigera* which is related to metabolism of protein and gossypol, CYP6AE1 from European parsnip which is related to insecticide resistance and the metabolism of exogenous compounds. It was followed by CYP6C, D, E, F, G, H and other family and lower homology with CYP1, 2, 3, 4, 5, 9 families.

**Table 1.**
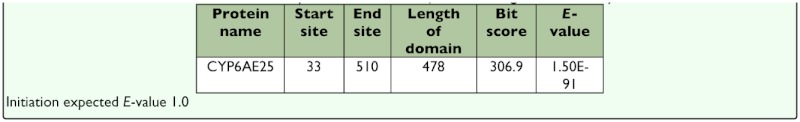
The functional domain of CYP6AE25 protein from *Ostrinia furnacalis* using Pham-line analysis.

### The developmental and tissue specific expression of *O. furnacalis CYP6AE25*

**Figure 11.**
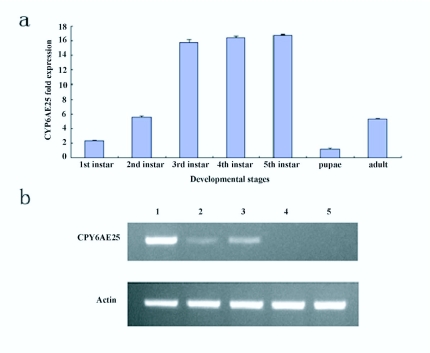
The mRNA transcripts level of corn borer, *CYP6AE25* gene. (a) The relative mRNA levels of *CYP6AE25* in different developmental stages. Quantitative analysis of mRNAs from 3-d-old 1^st^, 2^nd^, 3^rd^, 4^th^, and 5^th^ (last) instar larvae, pupae, and adults of *Ostrinia furnacalis*. Each bar represents the mean ± SD of four independent assays. (b) The transcript level of tissue specific expression of *CYP6AE25* using RT-PCR from 3^rd^ instar larvae. Lane 1 — midgut, Lane 2 — fat body, Lane 3 — Malpighian tube, Lane 4 — ovary, and Lane 5 — brain. High quality figures are available online.

Using two pairs of the primers, F1 and R1 for targeted cDNA, and F2 and R2 for internal reference, the quantitative real-time PCR was successful in amplification of a 234-bp size P450 cDNA fragment from the targeted cDNA template and a 262-bp size cDNA fragment from the internal reference. The relative expression pattern of mRNAs of *CYP6AE25* from 3 day old 1^st^, 2^nd^, 3^rd^, 4^th^, and 5^th^ (last) instar larvae, pupae, and adults is shown Figure 11a. It could be concluded that the *CYP6AE25* transcripts levels appeared in the order of 3^rd^ 4^th^ 5^th^ instar larvae > 2^nd^ instar larvae > adults > 1^st^ instar larvae > pupae. The mRNA levels in 1^st^ instar were 12.8 and 3 fold higher than those in pupae and adults, respectively. By RT-PCR detection, *CYP6AE25* in the midgut had a high level of transcription, while a small amount transcription level was observed in Malpighian tubes and fat body. The expression levels of *CYP6AE25* from 3^rd^ instar larvae appeared in the order of midgut, Malpighian tubes and fat body from high to low but absent in ovary and brain (Figure 11b).

## Discussion

Cytochrome P450 exists in all of the species, its involvement in a number of important life processes, and with the insect growth and development and on plant secondary metabolism has important biological and evolutionary significance (Bergé et al. 1998). The overexpression of many members of Cytochrome P450 families 6 and 9, such as *CYP6G1* and *CYP9A12* is associated with insect resistance. It is generally believed that insect P450 family members are involved in plant secondary metabolism and pesticide detoxification mainly due to increase in its mRNA and/or protein levels, resulting in mixed-function oxidase metabolism and detoxification enhancement. It has been confirmed in *M. domestica* and *D. melanogaster* and other insect species (Sudeshna and Roxanne 2001). Recently, the over-expression of *CYP6AE14* has been found in the midgut of *H. armigera* in the presence of gossypol (Mao et al. 2007; Fire et al. 1998). P450-mediated insect resistance mechanisms include the possibility of P450 over-expression and or amino acid residue change. The mutation of P450 regulation gene results in over-expression of P450, this may be the main mechanism of P450-mediated resistance. In the present study, *O. furnacalis CYP6AE25* has 52%, homology with *CYP6AE14* from *H. armigera* that is related to the metabolism of plant secondary substances. The *O. furnacalis CYP6AE25* P450 gene of P450 belongs to the CYP6 family. To date, P450 belonging to CYP6 family have been cloned from many insects such as the cotton bollworm, silkworm, housefly, fruit fly, cockroach and other insect species, and the majority of them are closely related to the metabolism of exogenous compounds such as plant toxins. The CYP6B1 from black swallowtail caterpillars, *Papilio polyxenes* was found to play an important role in the tolerance to toxic plant compounds (Li et al. 2001; Schuler 1996).

Cytochrome P450 monooxygenase has a number of functions related to the hemethiolate gene superfamily that plays a major role in the metabolism in a variety of endogenous and exogenous substances (Wang and Hobbs 1995; Daborn et al. 2002). Functional analysis is therefore needed to investigate whether *CYP6AE25* from *O. furnacalis* imparts tolerance to secondary metabolites. Down regulation of the expression of specific genes through RNA interference (RNAi) has been widely used for genetic research in insects. More significantly, expression of dsRNA directed against suitable insect target genes in transgenic plants has been shown to give protection against pests, opening the way for a new generation of insect-resistant crops. In summary, the identification of *CYP6AE25* in *O. furnacalis* provides information on the characteristics of P450s, which would be useful in future studies aimed at demonstrating the roles of P450s in insect tolerance to plant secondary metabolites and development of insect-resistant transgenic plants.
